# Integration of Low Intensity Psychological Support for Children and Young People Receiving Cancer Services: A Qualitative Study of Staff Perspectives

**DOI:** 10.1002/pon.70133

**Published:** 2025-04-02

**Authors:** Mariam Shah, Hannah Duncan, Helen Griffiths, Natasha Prescott, Rebecca Sweet, Isobel Heyman, Anna Roach, Anaïs d’Oelsnitz, Isabella Stokes, Zoe Berger, Sophie D. Bennett, Roz Shafran

**Affiliations:** ^1^ Great Ormond Street Hospital London UK; ^2^ University College London Great Ormond Street Institute of Child Health London UK; ^3^ St George’s, Epsom, and St Helier University Hospitals and Health Group London UK; ^4^ Institute of Psychiatry, Psychology & Neuroscience, King's College London London UK

**Keywords:** adolescent, child, cognitive behavioural therapy, mental health, oncology, psycho‐oncology, psychosocial intervention

## Abstract

**Objective:**

Despite children and young people (CYP) with cancer having elevated mental health needs, accessing evidence‐based psychological support remains difficult. Delivering low‐intensity cognitive behavioural therapy (LICBT) to CYP with cancer could increase access to support. This qualitative study aimed to understand the views of key clinician stakeholders regarding the potential facilitators and barriers to implementation of LICBT in paediatric cancer services.

**Methods:**

Semi‐structured interviews were conducted with 39 professionals working in paediatric hospital cancer services. Interviews were transcribed and analysed using framework analysis informed by the Consolidated Framework for Implementation Research.

**Results:**

Professionals indicated a potential need and utility for LICBT, and saw it as distinct from existing provision, addressing patient needs and filling a gap in services. Integration into the service and existing pathways was considered a key facilitator to successful implementation. Anticipated potential barriers included scepticism of LICBT efficacy due to the perceived high complexity of patient presentations and concerns about the relevance and suitability of existing manualized interventions for this population. LICBT practitioner ability to independently assess patient suitability for the intervention and to deliver LICBT effectively was also questioned.

**Conclusions:**

Cancer professionals' perceptions of LICBT indicate its potential use for the CYP cancer population to improve access to evidence‐based psychological interventions. Incorporating the identified facilitators and barriers into implementation strategies, including information about the effectiveness of LICBT for young people with chronic illness will help to ensure effective integration of LICBT into routine paediatric healthcare settings.

## 1 Background

In the UK, approximately 4200 children and young people (CYP) are diagnosed with cancer each year [1]. Over the past decade, childhood cancer survival rates increased from 77% to 84% [2]. Meanwhile, an increased psychological burden of the disease and its treatments has also been identified [3]. Nearly a quarter of childhood cancer survivors experience common mental health disorders [4, 5], which significantly worsen their health‐related quality of life outcomes [6]. Various evidence based psychosocial interventions effectively address mental and physical health symptoms for the paediatric oncology population [7]. National Institute for Health and Care Excellence (NICE) UK guidelines highlight the importance of supporting the psychological needs of CYP with cancer and their families [8, 9]. Yet, between 31% and 41% of CYP in the UK are unable to receive mental health interventions for their cancer diagnosis [10, 11] with 47% of those who did receive support reporting that they would be significantly negatively impacted if it were unavailable [11]. This unmet need is due to a lack of appropriate, available or affordable services and a lack of information about options for help [10]. One way to increase access to psychological interventions for this population is low intensity cognitive behavioural therapy (LICBT). Recommended as a first‐line evidence based treatment for common mental health problems for CYP in the general population, LICBT focuses on strategies to alleviate emotional distress and improve day to day functioning [12, 13]. Research indicates that CYP with long‐term health conditions have demonstrated improvements in mental health and quality of life following LICBT, with the intervention complementing existing psychological service provision in paediatric hospital settings [14]. LICBT typically consists of 6 h or less of contact time for up to 30 min delivered by trained pre‐qualification psychology professionals under senior clinicians' supervision [13]. If considered as the first line of action in paediatric cancer services, LICBT could lead to more timely support, decreasing waitlists and increasing capacity for senior clinicians to provide higher intensity interventions where needed. Overall, this would lead to less intrusive and time intensive interventions over time [15].

While LICBT can therefore be considered an evidence‐based intervention, it is not typically used within paediatric cancer services. As described by Bauer and colleagues [16], ‘The movement of evidence‐based practices (EBPs) into routine clinical usage is not spontaneous, but requires focused efforts’. Implementation science, ‘the scientific study of methods to promote the systematic uptake of research findings and other EBPs into routine practice, and, hence, to improve the quality and effectiveness of health services’ [16] can support such focused efforts. Implementation studies typically assess and implement strategies to improve uptake of services, and consideration of implementation from the start of the development of a new intervention pathway has been demonstrated to improve uptake and outcome [17, 18]. Typically, studies use frameworks that consider factors that may influence uptake at different levels. For example, the Consolidated Framework for Implementation Research (CFIR) is a practical framework to help guide systematic assessment of potential barriers and facilitators to guide tailoring of implementation strategies and needed adaptations [19]. It considers five domains: Innovation (the ‘thing’ being implemented, e.g., a new clinical treatment, educational programme, or service); Inner setting (the setting in which the innovation is implemented, e.g., hospital, school, city); Outer setting (the setting in which the Inner setting exists, e.g., hospital system, school district, state); Individuals (the roles and characteristics of individuals); Implementation process (the activities and strategies used to implement the innovation). Studies based on this framework therefore investigate the extent to which barriers and facilitators across these domains may influence uptake of an innovation. Typically, qualitative studies may be undertaken at the outset to understand key stakeholder views before implementation strategies are developed [20].

The present study aimed to use the CFIR as an implementation framework to understand the views of key clinician stakeholders regarding the potential facilitators and barriers to implementation of LICBT interventions in paediatric cancer services. It formed the first part of a planned overarching study to implement LICBT within a specialist paediatric cancer service setting.

## 2 Methods

### 2.1 Study Design

This qualitative implementation science study used a framework analysis approach to explore the barriers and facilitators of integrating LICBT into a specialist paediatric cancer setting. Semi‐structured interviews were conducted with healthcare providers from a single centre. Interview questions and data analysis were guided by the CFIR.

### 2.2 Setting

#### 2.2.1 Inner Setting

An existing specialist paediatric cancer setting with integrated mental health and wellbeing support provided by clinical psychologists.

#### 2.2.2 Outer Setting

A single specialist paediatric hospital in England hosted the paediatric cancer unit. LICBT had been previously running throughout the hospital as part of a drop in service study (young people attending the hospital, their parents and their siblings could ‘drop‐in/walk in’ to the centre and be provided LICBT if appropriate for their difficulty). The present paper reports part of a wider project to implement LICBT within routine practice.

### 2.3 Ethics

This project is part of a wider research study with approval granted by the London Riverside Research Ethics Committee (REC reference number: 16/LO/1915). The results informed the development of a service evaluation to evaluate the implementation of low intensity interventions in the same setting. Verbal consent was received from participants in line with these approvals.

### 2.4 Participants

Fifty‐five professionals working in, or closely aligned to, cancer services in a specialist paediatric hospital in the UK were approached for interview. Thirty‐nine professionals took part (Table 1), including three focus groups and 22 individual interviews. Those that did not consent felt that this was not applicable to their role or were unavailable. The mean age was 41 years, the majority identified as female (female: 27; male: 8; unknown: 4) and White British (*n* = 18). The average number of years working in the current service was 7, and the average number of years since qualifying in their profession was 15.

**TABLE 1:** Participant characteristics.

Profession	*N* =
Clinical psychologist	10
Medical consultant	9
Nurse	10
Other (e.g. play specialist, physiotherapist, assistant psychologist)	10

### 2.5 Participant Selection

Professionals were purposively sampled to provide insight into the patient population and existing service provision. Participants were identified through remote multi‐disciplinary team meetings and invited by email to interview. Snowball sampling occurred when participants identified other professionals appropriate for an interview. Sample size was determined by the number of professionals in the service that consented to interview [21].

### 2.6 Data Collection

Semi‐structured interviews were conducted between July and October 2023 by M.S. (female, assistant psychologist and senior research assistant, with prior experience of qualitative research), I.S. (female, senior educational mental health practitioner and research assistant, with prior experience of qualitative research) and facilitated by A.d’O. (female, research assistant, new to qualitative research). None of the interviewers had pre‐existing relationships with the participants.

The interview guide was developed by A.R., R.S. and S.B. (Supporting Information S1) and was guided by the CFIR. Participants were first provided with a definition of LICBT and previous research findings demonstrating the efficacy of LICBT in CYP with a chronic health condition populations [14]. Participants were told that subsequent plans to implement LICBT in the service would be informed by the present study findings. They were then asked questions on the interview schedule, which was developed considering the CFIR domains. In line with the CFIR, it included questions regarding the current mental health provision including asking what participants considered to be the similarities with, differences to, benefits of, and limitations of, LICBT. Questions also helped to identify facilitators and barriers to implementation across the five CFIR domains. Interviews were 30–60 min, completed online or in person and were all recorded. Staff were interviewed either individually or in a focus group. Verbatim transcripts were generated and pseudo‐anonymised.

### 2.7 Data Analysis

A framework analysis was conducted including: transcription; familiarisation; coding; application to analytical framework; charting into framework matrix; interpretation. M.S. was the primary coder and completed data analysis steps with input from senior researchers (S.B. and R.S.). All transcripts were read thoroughly and a reflective journal was recorded to discuss reflexive notes and initial impressions. Initial codes were then generated using qualitative software NVivo 12. Transcripts were analysed deductively using the CFIR domains which formed the analytical framework. The CFIR was chosen to help identify potential facilitators, barriers and strategies for successful implementation of LICBT in a paediatric hospital setting [19]. The five CFIR domains are: Innovation (including how it was similar or different to existing provision); Inner setting; Outer setting; Individuals; Implementation process. The data was charted into a framework matrix and interpreted through an iterative process to develop the final themes. To maintain validity, several strategies were employed including reflective journaling, supervisory guidance and sensitivity to context, rigour and recognition of impact [22].

## 3 Results

### 3.1 Main Findings

Professionals' perspectives on the implementation of LICBT in the paediatric hospital cancer service resulted in feedback across all five CFIR domains.

### 3.2 Innovation

The innovation refers to the characteristics of the LICBT intervention. While psychology professionals had some initial knowledge of LICBT, the majority of non‐psychology professionals were unfamiliar with these interventions prior to interview.

#### 3.2.1 Knowledge About the Innovation

Most professionals believed that LICBT differed from existing psychological support in the hospital setting and explained that they were unaware of it being routinely employed. Types of intervention currently offered focused on end of life and bereavement, acceptance of/adjustment to diagnosis, procedural anxiety, behavioural interventions, needle phobia, low mood and low self‐esteem. The type of psychological intervention delivered was tailored to patient needs, influenced by factors such as patient and family requests, patient age and contextual considerations like access requirements. Some identified similarities between LICBT and interventions that were currently provided (e.g., considering group work that was offered to be low intensity). The use of manuals was seen as distinct from the existing interventions.

There is definitely a range, we currently do some group workshop stuff which could be classed as low intensity, we do signposting, we do sessions of anxiety work. I think people do it [low intensity work] and don’t realise they’re doing it.

(HCP6)

### 3.3 Inner Setting

The inner setting refers to the existing psychological service located within the cancer service.

#### 3.3.1 Compatibility With Existing Psychological Services

Professionals described LICBT as compatible with the existing psychological service and stepped care model approach but suggested that it would not be suitable for acutely unwell patients or those with severe mental health problems. Professionals suggested that LICBT should be offered alongside an array of existing options for psychological support. Some suggested that LICBT might be suitable to offer to siblings to address the gap in support in existing provision.

Several non‐psychology professionals thought that any form of psychological intervention should be offered to all patients diagnosed with cancer ‘*rather than waiting for someone to cry for help*’ (HCP12).

#### 3.3.2 Available Resources

Capacity for additional psychological intervention was seen as limited, and while some considered existing staff to be suitable LICBT practitioners, others thought that new staff would need to be employed. Concerns about suitable space availability for LICBT delivery were raised, with remote delivery presenting a potential solution.

### 3.4 Outer Setting

The outer setting in this context refers to the wider structure of the hospital trust, the National Health Service (NHS) and external agencies connected to hospital services.

#### 3.4.1 Funding and Resources

Professionals were concerned that limited funding and resources NHS‐wide would not be able to support the implementation of the innovation, meaning external set funding would be required.

#### 3.4.2 Partnerships and Connections With External Support Agencies

Existing services were perceived to offer general ‘low intensity’ support through partnerships with external support agencies such as social work, hospices and community services. Psychology professionals were clear that LICBT should not overlap with such support already offered externally. Referrals or signposting to these agencies were based on patient needs and circumstances, with some accessing this support sooner and others later, depending on community support wait times.

Our psychologists will often refer to local CAMHS services, and then the patients end up waiting for months, years for CAMHS to see them. Whether there is low level support that they could get in that period, it would be useful.

(HCP5)

### 3.5 Individuals

#### 3.5.1 Innovation Practitioners

Various professions were suggested as suitable LICBT practitioners depending on individual families' preferences and needs. Clinical psychologists, trainees and assistant psychologists were frequently recommended for their knowledge and skills, or nurses and clinical nurse specialists (CNS) due to their close relationship with, and understanding of, families.

Considerations for LICBT practitioners included capability, motivation and opportunity. One professional suggested that training non‐psychology professionals to deliver LICBT could result in ‘*kind of blurring of lines between professions that I think might make their jobs a bit more challenging*’. (HCP10). Stakeholders highlighted staff capacity would limit opportunity to deliver LICBT, particularly for psychologists, assistant psychologists, nurses, CNS and play specialists.

### 3.6 Implementation Process

The implementation process refers to strategies devised in collaboration with professionals from the inner setting that would support successful implementation of the innovation.

#### 3.6.1 Facilitators for Successful Implementation

Streamlining integration of the LICBT into existing hospital psychology services and referral pathways was identified as a key facilitator required for successful implementation. This included integration of LICBT practitioners into the service and that existing staff understood LICBT interventions. Mindful use of language and clear communication of what the LICBT intervention involves, and who was suitable to refer, for both professionals and patients was also deemed important.

I guess I'm thinking about how to kind of make things as streamlined as possible and it feels like that intervention would need to be part of what [the existing psychology team] offers.

(HCP18)

#### 3.6.2 Barriers to Implementation

Several barriers to implementation were identified, including scepticism of LICBT intervention efficacy, and concerns about its manualized nature. This included a perceived lack of flexibility and an inability to adapt to cancer patients' specific needs and complex presentations. Additional concerns related to the ability of LICBT practitioners to independently assess patient needs and effectively deliver LICBT, and the potential to overlap or undermine existing psychological support provision. Another barrier was families' limited capacity to engage with multiple physical, mental health and research‐related appointments.

I'm not quite sure how that's going to work in terms of the manualised approach in terms of the psychosocial difficulties that these young people are experiencing.

(HCP27)

#### 3.6.3 Tailoring Implementation

Stakeholders held different views regarding when to offer LICBT. Many professionals suggested introducing LICBT at patient diagnosis to provide support at this difficult time and establish a long‐term relationship with relatively few sessions. Conversely, others were against offering LI at diagnosis due to families being overwhelmed and needing time to process new information. Stakeholders noted increased difficulties were often experienced at the end of treatment or during long‐term follow‐up due to less support generally being offered at this time, despite patients and their families typically having a greater ability and capacity to engage. Some deemed LICBT unsuitable for those on active treatment, while others emphasised a need for ongoing flexible integration of LICBT based on the families' needs and medical treatment experience.

#### 3.6.4 Assessing Staff Needs

Stakeholders emphasised the need for bespoke training addressing the application of LICBT to paediatric cancer and unique challenges this presented.

Clinical supervision was deemed crucial for creating a supportive, safe, consistent space for LICBT practitioners to discuss patients, receive feedback and safeguard their mental wellbeing. Supervisors would need skills in LICBT models, an understanding of the present psychology services and hospital context, and the ability to support formulation and determine which patients are suitable for the intervention. Some professionals suggested utilising existing clinical psychologists as supervisors for better integration, depending on team members' existing capacity and capability to supervise LICBT, rather than employing staff external to the cancer service.

#### 3.6.5 Engaging Staff With the Innovation

Most professionals believed everyone involved in patient care could benefit from education in LICBT (i.e., what it is, who it is suitable for, how to refer), though some suggested that only those referring patients would benefit. Concerns were raised about potential confusion among medical professionals regarding different types of psychological support, highlighting the need for such training.

#### 3.6.6 Evaluating the Innovation

Professionals emphasised the importance of collecting subjective measures like goal‐based outcomes to evaluate the long‐term outcomes of LICBT. Other suggestions included hospital derived data, service level measures or psychological measures such as the Strength and Difficulties Questionnaires (SDQ) [23] and Generalised Anxiety Disorder scale (GAD‐7) [24]. However, they expressed caution about the use of measures that might not be sensitive to health‐related issues, as well as the possibility of measures increasing patient burden. Patients, their families, and professionals involved in their care were recommended to provide feedback.

## 4 Discussion

This research aimed to investigate facilitators and barriers to the planned implementation of LICBT within a paediatric cancer setting to address a gap in provision for the mental health needs of CYP with cancer. Overall, facilitators for successful implementation included a level of clinician ‘buy‐in’—they considered that there was a need for more support and that LICBT may be suitable for the needs of CYP with cancer. However, key barriers included scepticism of LICBT efficacy and suitability for CYP with cancer, including concerns about the use of manualized interventions. Specifically, several stakeholders were concerned that LICBT would not effectively address patient needs due to perceived complexity of patient presentations; patients with emotional needs resulting from physical illness or treatment are often perceived to require different interventions from the evidence‐based interventions shown to be effective in young people without illness [25]. Research does show LICBT's efficacy in alleviating psychological symptoms for CYP with long‐term health conditions [14] and reducing cancer‐specific distress for patients and caregivers [26]. Similarly, concerns about the limited flexibility of manualized interventions for CYP with cancer are addressed in research that demonstrates fidelity to LICBT protocols while allowing for flexibility through adaptation to individual needs [27]. However, these stakeholders would be unlikely to fully buy into any implementation at present and thus a strategy would need to be devised to bring them on board with LICBT being offered in the service. For example, the stakeholders may benefit from further training in how LICBT can be tailored to meet the needs of CYP with cancer. Alternatively, or additionally, further research or a staged implementation process may need to be conducted to evaluate its use in a small number of patients, potentially modifying the intervention as needed, before being implemented more widely [28].

Similarly, there were also concerns about who would deliver the intervention and whether they would have adequate supervision. Some professionals felt that existing qualified, trainee and pre‐qualified psychologists, nurses and Clinical Nurse Specialists familiar with the inner setting would be suitable practitioners. Staff selection for this role must be considered in relation to the current nursing recruitment crisis restricting capacity to train in and deliver LICBT in addition to existing duties [29]. With staffing being a key facilitator (and poor staffing presenting a key barrier) to implementing LICBT in routine care settings [30] these recommendations must be considered within the context of the changing healthcare landscape and a drive to innovate through utilisation of existing resources [31]. However, stakeholders also noted the need for adequate supervision and questioned whether there was capacity in the service for this, both in terms of time but also expertise. It is likely that training would need to be delivered to supervisors in the first instance to ensure that supervisors had the expertise perceived to be required. A recent study undertaken by our team implemented an approach in which a CBT intervention for children with epilepsy was iteratively modified with stakeholder input, then training was developed, and finally the resultant training and intervention were piloted and trialled [32]. A similar approach may be warranted for LICBT in cancer services.

Once such barriers are overcome, what would an LICBT pathway developed with implementation in mind look like? Ensuring that LICBT was integrated into existing service systems rather than a stand‐alone offering was considered important, together with clear communication with stakeholders about LICBT. In line with this, previous research has discussed the importance of integrating mental health interventions into existing psychology team pathways and systems [33]. Coordinated care pathways help alleviate patient worries and ensure treatment completion [34]. LICBT could be part of a stepped care or staged care model, depending on existing service pathways. LICBT's potential utility as an initiative to reduce long wait lists for intervention was also recognised, given the challenges in accessing mental health support both in the inner setting and nationwide [35].

Professionals had different views on when LICBT should be offered to patients. While many favoured introducing LICBT at diagnosis due this being a highly distressing time for patients [36], others raised concerns about overwhelming families with new information at this time [37]. Initial research suggests that internet administered LICBT is feasible and acceptable to parents of children with cancer, potentially addressing concerns about families' capacity to engage with the intervention [38]. End of cancer treatment and long‐term follow‐up were also deemed suitable times for introduction to LICBT aligning with NICE guidelines recommendation for sustained psychological support post cancer treatment [8]. Professionals felt only a minority of CYP access support at the end of treatment [11] and echoed concerns about patients experiencing unprocessed emotions and difficulties in readjusting to ‘normal’ life at this time [39]. Considering the variety of recommendations for when to introduce LICBT, future research should incorporate CYP and family perspectives to determine the most meaningful time [40].

### 4.1 Study Strengths and Limitations

Key strengths to this study include the large sample size, originality of the research and importance of the question. While the interview participants had experience working with CYP with cancer and an understanding of the hospital infrastructure, most were non‐psychology professionals, and it was not always clear that they understood what LICBT was, and how this differed from other kinds of psychology support. Including more detailed examples of interventions available through LICBT at the start of focus groups and interviews may have helped all participants, including the psychologists, to have answered in a more informed way. Due to the present study being conducted in a single centre specialist paediatric cancer setting with embedded psychological support, study findings may have limited generalisability, particularly considering the variability of wider existing integrated care for this population [11]. Other services seeking to implement LICBT for CYP receiving cancer services must consider facilitators and barriers to implementation given the individual service context and service user needs. The study would have benefitted from the inclusion of young people with cancer and their families to understand their perspectives on the implementation of LICBT.

### 4.2 Clinical Implications and Conclusion

This study highlights the potential facilitators and barriers to the implementation of LICBT in CYP with cancer, with the desired outcome of increasing access to and provision of evidence‐based psychological support for this population. While clinicians recognised a need and role for LICBT, a number of barriers need to be addressed before LICBT can be implemented routinely within paediatric cancer services. These include scepticism regarding the efficacy of LICBT in this population and availability of adequate resource to conduct and supervise the work. A stepped approach to implementation may be beneficial in the first instance to allow such concerns to be iteratively addressed.

## Author Contributions

**Mariam Shah:** methodology, formal analysis, writing – original draft, writing – review and editing, project administration. **Hannah Duncan:** supervision, writing – review and editing. **Helen Griffiths:** conceptualisation, methodology, writing – review and editing, funding acquisition. **Natasha Prescott:** writing – review and editing. **Rebecca Sweet:** conceptualisation, methodology, writing – review and editing. **Isobel Heyman:** conceptualisation, writing – review and editing, funding acquisition. **Anna Roach:** conceptualisation, writing – review and editing, funding acquisition. **Anaïs d’Oelsnitz:** formal analysis, writing – review and editing. **Isabella Stokes:** conceptualisation, writing – review and editing. **Zoe Berger:** conceptualisation, methodology, funding acquisition, writing – review and editing. **Sophie D. Bennett:** conceptualisation, methodology, formal analysis, writing – original draft, writing – review and editing, supervision, project administration, funding acquisition. **Roz Shafran:** conceptualisation, methodology, formal analysis, writing – original draft, writing – review and editing, supervision, project administration, funding acquisition.

## Conflicts of Interest

The authors declare no conflicts of interest.

## Supporting information

Supporting Information S1

## Data Availability

The authors have nothing to report.
