# Supplementary material for: Integration of Low Intensity Psychological Support for Children and Young People Receiving Cancer Services: A Qualitative Study of Staff Perspectives
Source: Psychooncology. 2025 Apr 2;34(4):e70133. doi: 10.1002/pon.70133 (PMC11964891; doi:10.1002/pon.70133)
Supplement: Supplementary file 1 — Supporting Information S1 [file PON-34-e70133-s001.docx]

**Healthcare professionals’ experiences and views of low intensity interventions for children with cancer**

**Topic guide for Semi Structured Interviews:**

1) Current mental health provision

- 1. What is the usual care pathway for children with cancer with mental health needs? (prompts below)
  2. What psychological treatment is currently provided?
  3. Does this differ by diagnosis?
  4. Does this differ by treatment?
  5. Does this support differ depending treatment stage (e.g. active chemo or in remission)
  6. What are the current waits for treatment?
  7. What support (if any) is offered to the wider family (Parents and siblings)

1. Thinking now about low intensity interventions
   1. What do you know about low intensity interventions?
      1. E.g. guided self help (we would provide definition/examples)
   2. Is this/does this sound different to what Is currently provided?
   3. How might this work in your service?
   4. How does this fit with other existing services
   5. Where in the care pathway might this fit? (e.g. some teams have found guided self help as a useful waiting list initiative…)
2. How might low intensity CBT help families in your service? Do you think this might be successful in practice?
3. Thinking now about implementation…

If low intensity intervention were to be implemented, what is important to consider in your team? (it might be helpful to think about when other new services have been implemented)

- 1. Which health care professionals would need to support this?
  2. What financial factors should we consider?
  3. Which workforce could deliver these interventions? who is in the best place?
  4. Support from colleagues/ management
  5. How could we increase people’s understanding or views of low intensity CBT

1. What might the training needs be for staff (we have 2 days of recorded training)
   1. Mental health or non mental health staff?
   2. Training duration
   3. Ongoing supervision (by psychologists)
2. Outcome measures
   1. How would you know if this is successful?
   2. How would we track progress? (what is used currently?)

1. Any final words or anything to share about low intensity CBT?
